# Editorial: T-Cell Migration in Health and Disease

**DOI:** 10.3389/fimmu.2017.00132

**Published:** 2017-02-13

**Authors:** Vinicius Cotta-de-Almeida, Loïc Dupré, Wilson Savino

**Affiliations:** ^1^Laboratory on Thymus Research, Oswaldo Cruz Institute, Fiocruz, Rio de Janeiro, Brazil; ^2^UMR 1043, Centre de Physiopathologie de Toulouse Purpan, INSERM, Toulouse, France; ^3^Université Toulouse III Paul-Sabatier, Toulouse, France; ^4^UMR 5282, CNRS, Toulouse, France

**Keywords:** T cells, migration, motility, immunity, endothelial cells, antigen-presenting cells

**Editorial on the Research Topic**

**T-Cell Migration in Health and Disease**

The trafficking of leukocytes is a key process to allow and regulate their immunosurveillance duties. Indeed, the high motility capabilities of immune cells are coupled to their ability to detect and eliminate pathogens and tumors. These motility capabilities are also coupled to the development of unwanted responses against autologous or grafted tissues. Trafficking throughout the organism and local motility within tissues has direct ontogenetic and functional impacts on lymphocytes, including maturation and differentiation of effector and regulatory T cells. Ultimately, the tuning of T cell migratory activity leads to the coordinated antigen scanning of T cells along their paths from blood to lymphoid organs and to peripheral non-lymphoid tissues (1).

Despite the advances for understanding the molecular and cellular bases of the complex process of T-cell migration, only recently the mechanistic rules that operate the migratory patterns at the several stages of T-cell development and activation have been depicted in detail. Moreover, the new imaging technologies unravel the role played by the several molecules that regulate the T-cell shape remodeling underlying motility, the initial interaction with antigen-bearing cells and the final decision to act on an effector immune response or on its regulation (2).

In this context, the goal of this research topic was to bring together a collection of thoughtful papers that approach T-cell migration in physiological and pathological conditions, highlighting the molecular regulation and the dynamics of T-cell motility, the central cellular interactions, and the distinct tissue microenvironmental cues, which, altogether, are critical for the outcome of T-cell-mediated immune responses.

As T cells migrate to distinct tissue compartments in the search for antigens, endothelial cells act as partners for cell–cell interactions, and such contacts serve as a major regulation of the T-cell trafficking process. This topic is elegantly approached by Carman and Martinelli in a review describing such interaction as an event that directly influences T-cell activation and differentiation. This regulatory role is emphasized from a thorough description of the unique properties of endothelial cells as “semiprofessional” non-hematopoietic antigen-presenting cells (APCs), as they mediate Ag-specific stimulation of Ag-experienced T cells and further control the transmigration process of effector cells.

The molecular dynamics encompassing the central process of antigen scanning by T lymphocytes on the surface of APCs, and the consequent decision for T lymphocytes to migrate or to stop migration is directly discussed in two papers. Stein brings a perspective article that discusses the molecular control of the T-cell motility in the activation and effector stages of the immune response. He points out the results from *in vivo* studies applying two-photon microscopy, revealing the dynamics of these events and also that molecules involved in membrane–membrane interactions, as well as the fact that chemokines (CCR5 ligands) and the lipid mediator thromboxane A2 are active players in regulating both T-cell migration and the formation of T cell–APC contacts. Dupré et al. explore, in a review paper, the molecular regulation of actin cytoskeleton remodeling in T cells. This review comprehensibly integrates the several studies on the dynamics of T-cell motility, pointing how the remodeling of the actin cytoskeleton drives the migration of T cells, allowing them to cross endothelial barriers and further interact with the tissue extracellular matrix (ECM) meshwork and with antigen-bearing cells.

Critical microenvironmental cues that trigger T-cell migration, such as ECM ligands and chemokines are also reviewed in this research topic. Savino et al. discuss the interplay of the migratory activity of developing T cells with their complex process of intrathymic differentiation, pointing out the ECM protein laminin (LM) and their integrin-type receptors as central players in the multivectorial model that describes the intrathymic thymocyte migration. This review also poses an interesting debate about the LM-mediated interactions as a vector that regulates and is regulated by other vectors, such as the semaphorin/neuropilin and CXCL12/CXCR4 interactions, respectively. Karin and Wildbaum present a mini-review that focuses on chemokines as drivers for polarization of the T-cell effector function as well as for differentiation of regulatory T cell subsets. In addition, they discuss the clinical implications of proposed chemokine-based therapy for autoimmune and neoplastic diseases.

A further topic that is discussed is the migration of effector T lymphocytes, such as CD8^+^ cytotoxic T cells. In the context of an immune response to tumors, T cells migrate to the tumoral tissue, contact tumor cells, and associated APCs, and perform an intra-tissue migration. The original research article from Bougherara et al. describe a technique that employs real-time imaging microscopy to track the migratory properties of fluorescent CD8^+^ T cells plated onto slices of a carcinoma biopsy. Their results describe speed, direction, and distribution of these cells within the tumoral tissue and the modulation of these migratory activities by microenvironmental components, such as collagen fibers.

Braiman and Isakov address the regulation of both T-cell adhesion and migration exerted by Crk adaptor proteins. The authors describe, in a mini-review, the structural and biochemical properties of these proteins, including their integration in distinct signaling pathways, which regulate T-cell adhesion to tissue ECM and the chemokine-driven migration to inflammatory sites.

The migration of effector T cells toward sites of effector function in autoimmune diseases is also covered herein. Mellado et al. compile several studies on T-cell migration in the context of the autoimmunity in rheumathoid arthritis. This review highlights the mechanisms involved in T-cell migration from blood to the synovium and the resulting effector function in this inflammatory site. Likewise, it discusses that chemokines and their receptors can be placed as therapeutic targets in the rheumathoid arthritis.

In summary, the eight articles composing this research topic provide insights into key and complementary mechanisms underlying the complex process of T-cell migration, both in physiological and pathological conditions.

## Author Contributions

VC-d-A, LD, and WS contributed equally to this editorial article.

## Conflict of Interest Statement

The authors declare that the research was conducted in the absence of any commercial or financial relationships that could be construed as a potential conflict of interest.
